# Impact of Zinc Oxide Nanoparticles on the Composition of Gut Microbiota in Healthy and Autism Spectrum Disorder Children

**DOI:** 10.3390/ma14195488

**Published:** 2021-09-23

**Authors:** Rongrong Yu, Temoor Ahmed, Hubiao Jiang, Guoling Zhou, Muchen Zhang, Luqiong Lv, Bin Li

**Affiliations:** 1 School of Education Science and Technology, Zhejiang University of Technology, Hangzhou 310032, China; rrongyu@126.com; 2 Institute of Biotechnology, Zhejiang University, Hangzhou 310058, China; temoorahmed@zju.edu.cn (T.A.); 371112@zju.edu.cn (H.J.); 11816060@zju.edu.cn (M.Z.); 22016087@zju.edu.cn (L.L.); 3 Hangzhou Seventh People’s Hospital (HSPH), Hangzhou 310013, China; zhougl0120@163.com

**Keywords:** ASD, bacterial numbers, community structure, gut microbiome, nanoparticle resistance

## Abstract

Autism spectrum disorder (ASD) seriously affects children’s health, while the gut microbiome has been widely hypothesized to be involved in the regulation of ASD behavior. This study investigated and compared the number, diversity, and population structure of gut microbiota between healthy and ASD children and their susceptibility to zinc oxide nanoparticles (ZnONPs) based on the measurement of live cell number, living/dead bacterial staining test, flow cytometry observation and bacterial community analysis using 16S rRNA gene amplicon sequencing. The result of this present study revealed that ASD children not only significantly reduced the live cell number and the community diversity of gut bacteria, but also changed the gut bacterial community composition compared to the healthy children. In addition, this result revealed that ZnONPs significantly reduced the number of live bacterial cells in the gut of healthy children, but not in that of ASD children. In contrast, ZnONPs generally increased the gut bacterial community diversity in both ASD and healthy children, while a greater increase was found in ASD children than that of healthy children. Furthermore, this study successfully isolated and identified some representative nanoparticle-resistant bacteria based on the color, shape, and edge of colony as well as the 16S rDNA sequence analysis. The community of nanoparticle-resistant bacteria differed in between healthy and ASD children. Indeed, the representative strains 6-1, 6-2, 6-3 and 6-4 from healthy children were identified as *Bacillus anthracis*, *Escherichia coli*, *Bacillus cereus* and *Escherichia coli* with sequence similarity of 97.86%, 99.86%, 99.03% and 99.65%, respectively, while the representative strains 8-1, 8-2 and 8-3 from ASD children were identified as *Bacillus cereus*, with sequence similarities of 99.58%, 99.72% and 99.72%, respectively. Overall, this study demonstrated that ZnONPs caused a change in number, diversity, and species composition of gut bacteria, but differed in healthy and ASD children.

## 1. Introduction

Autism spectrum disorder (ASD) is a severe neurodevelopmental disorder characterized by abnormal social interactions, impaired language, and stereotypical and repetitive behaviors [1,2,3,4]. In the last decade, more and more children have been diagnosed with ASD, which affects approximately 8–12% of children school-aged children worldwide [1,5]. Furthermore, the number of ASD children has also significantly increased in China in the last few years [2]. Nowadays, ASD is becoming a public health concern with an incidence rate of about 0.26% in Chinese children. Although pharmacological treatments are able to reduce the behavioral problems, they are often found to be ineffective in treating the core symptoms of ASD (i.e., social deficits). Furthermore, Nogay and Nahikian-Nelms reported that most of the randomized clinical trials of ASD deficits treatments have shown inconclusive results because of clinical and genetic heterogeneity [6].

There is an increasing incidence of ASD, however, the pathogenic mechanisms underlying ASD is not well understood. Many studies have focused on finding the genetic causes of ASD, which have been reported to be related with the disease, but only 1/3 of all autistic cases were able to be explained by genetic causes [7]. However, the continued increase of ASD incidence at a rate makes it necessary for us to correlate this disease with non-genetic factors, such as pregnancy and maternal-related factors [8]. Furthermore, some studies have reported that ASD can be caused by various factors, i.e., genetic factors, immune dysregulation, comorbid disorders of the central nervous system, and an aberrant innate immune response against endotoxins; however, there is no clear evidence to prove these claims. Nowadays, it is well known that a single cause is unable to account for all ASD-related behaviors, while increasing evidence has shown that the causes should be attributed to multifactorial etiologies such as gene-environment interactions, environmental factors, de novo mutations, genetic risk factors, perinatal events and utero exposures [9,10,11].

Recently, more and more attention has been paid on the role of the human gut microbiome in the development and severity of ASD. The intestinal tract of humans has been colonized by thousands of bacterial species during the coevolution of man and microbes. This will give us a better clue to reveal the etiology of this multifaceted disease and open the possibility for efficient treatment of ASD patients. Interestingly, as the largest micro-ecosystem in human body, the intestinal microbiota has been found to have an influence in brain function and social behaviors through involvement in immunological development, physiological balance, amino acid metabolism, and glutathione metabolism [12,13,14,15]. Indeed, several studies showed evidence of changes in microbial community structure by comparing the composition of gut microbiota between healthy and ASD children [16,17,18]. A number of gut bacteria have been proposed to be highly associated with ASD, however, bacterial species differed in different studies [16,17,18].

On the other hand, there was increasing evidence that green synthesized nanomaterials have a great potential to be used as therapeutic agents [19]. Furthermore, few experimental studies have been performed on the effect of metallic nanoparticles on intestinal microbiome/microbiota [20]. Indeed, previous studies have revealed the bactericidal effect of green synthesized metallic nanoparticles against human pathogenic Gram-negative bacteria *Escherichia coli* and Gram-positive bacteria *Staphylococcus aureus* [21]. The conflicting results from different studies should be attributed to the difference in the kind, incubation time and concentration of the tested nanoparticles. Furthermore, recent studies found that the number, diversity and species community of gut bacteria were able to be affected by both ADHD (another kind of childhood neurodevelopmental disorder) and bio-synthesized ZnONP [22,23]. Previous studies showed that ZnO nanocomposites have selective toxicity toward normal and cancerous cells, however, biological methods of synthesizing ZnO nanoparticles seem to be environmentally friendly and much safer than the physical and chemical approaches [24]. Therefore, it was hypothesized that the bio-synthesized ZnONP was also able to reduce the diversity of gut bacteria from ASD children.

In order to examine the effect of nanoparticles in gut bacteria, this research examined and compared the number, diversity and composition of intestinal microbiota between healthy and ASD children as well as the influence of ZnONPs on intestinal microbiota by a comprehensive analysis, including bacterial colony counting method, living and dead bacterial cell staining, and flow cytometry test as well as analysis of the 16S rDNA sequence and 16S rRNA gene amplicon sequence.

## 2. Materials and Methods

### 2.1. Collection of Fecal Samples

Twenty-four ASD children with the mean age of 6.2 years were recruited in this study, while the diagnose and treat of these ASD children were carried out between January 2019 and July 2020 in the Pediatric Department of Hangzhou Seventh People’s Hospital, China. On the other hand, we recruited thirty-eight healthy children with the mean age of 8.6 years from primary schools, Hangzhou, which were used as the control group in this study. The identity of ASD children (between 3 to 12 years old) was determined according to the method of DSM-5 (Diagnosis and Statistical Mannual of Mental Disorders-5th edition). Afterward, the fecal samples were kept at −20 °C for analysis of intestinal microbiota.

### 2.2. Mixture of Fecal Samples

In order to increase the reliability of data, all measurements in this study were carried out on the mixed fecal samples. In brief, about one gram fresh feces from each ASD children was dissolved into 10 mL sterile deionized water and shaken and mixed thoroughly. Following centrifugation of fecal solution at 10,000× *g* for 3 min at 4 °C, the mixed fecal sample of ASD children were obtained by collecting the pellets and then storing at 4 °C for further use. Furthermore, the mixed fecal sample of healthy children was prepared by using a similar procedure.

### 2.3. Effect of ZnONPs on Gut Microbiota

In order to examine the impact of ZnONPs on gut microbiota, the mixed feces from both ASD and healthy children were dissolved in distilled water, respectively, afterwards, the solution of the mixed feces was added with the stock solution (1.0 mg/mL) of ZnONPs up to obtain a final density of 20 µg/mL. Biogenic ZnONPs of spherical shapes with particle size ranging from 22 to 46 nm was provided by the Institute of Biotechnology, Zhejiang University, Hangzhou, P.R. China, which was biosynthesized using *Acinetobacter johnsonii* strain RTN1 and characterized in our recent study [25].

### 2.4. The Traditional Plate Counting Method

The surviving bacterial cells were enumerated by the plate counting method, which was carried out as described by Li et al. [26]. In brief, the mixed feces from ASD and healthy children were dissolved in distilled deionized water, while the impurities were removed by filtration with four layers of gauze. Following the 10-fold serial dilution of gut bacterial suspensions and inoculation of 10 μL samples on nutrient agar medium at 37 °C for 48 h until visible colony formation, the cell numbers of gut bacteria was determined by counting the colony number on the nutrient agar medium and then calculating the mean value of the colony number at the lowest dilution. Each treatment consists of six replicates and this experiment was repeated twice.

### 2.5. Living and Dead Bacterial Cell Staining

The damage of 20 μg/mL ZnONPs to the cell membranes of gut bacteria was determined as described in our previous studies [25] by using the method of living and dead bacterial cell staining, which was carried out based on the protocol of the BacLight bacterial viability kit (Invitrogen) following the induction of Isopropyl-beta-D-thiogalactopyranoside (IPTG) with a final density of 1 mM. The kit consists of two nucleic acid stains, which was named (i) green fluorescent (SYTO 9 stain) for live bacteria and (ii) a red-fluorescent (propidium iodide stain) for dead bacteria. An Olympus inverted confocal microscope was used to observe the fluorescence of gut bacteria in the mixed fecal samples from ASD and healthy children in the presence and absence of ZnONPs.

### 2.6. Flow Cytometry Assay

The damage of 20 μg/mL ZnONPs to gut bacteria were further determined by calculating the live and dead bacterial cells in the mixed fecal samples from ASD and healthy children in the presence and absence of ZnONPs, which was carried out using flow cytometry assay as described by Pagano et al. [27] with minor modification. Briefly, following the conventional IPTG induction for 12 and 24 h, and centrifugation at 5000× *g* for 10 min, the bacterial pellets were harvested, then washed three times using distilled deionized water, and finally re-suspended in the PI solution of 50 mg/L. After staining the bacterial cells for 20 min in dark, then washing three times, the staining bacterial cells were observed using flow cytometry (Beckman Coulter, Gallios, Germany).

### 2.7. Analysis of 16S rRNA Gene Amplicon Sequence

The effect of 20 μg/mL ZnONPs on the gut microbiome was determined by examining the change in gut bacterial community structure based on 16S rRNA amplicon sequence analysis, which was performed as described by Zhou et al. [25]. In brief, total genomic DNAs was extracted from the mixed fecal samples using the DNA Kit (D5625-01, OMEGA Bio-Tek, Norcross, GA, USA) following the manufacturer’s protocol, and then detected by NanoDrop ND-1000 spectrophotometer (Thermo Fisher Scientific, Waltham, MA, USA). After PCR amplification using forward/reverse primers 338F/806R, purification by Vazyme VAHTSTM DNA Clean Beads (Vazyme, Nanjing, China), and quantification using Quant-iT PicoGreen dsDNA detection kits (Invitrogen, Carlsbad, CA, USA), The PCR products were used to construct a DNA library following the 16S rRNA Sequencing Library Preparation instructions (Illumina, CA, USA). The amplicons sequencing were carried out on the MiSeq platform (Illumina, CA, USA) with the MiSeq Reagent Kit v3 (600 cycles) from Shanghai Personal Biotechnology Co., Ltd. (Shanghai, China). Sequence data were analyzed using a series of bioinformatics tools including QIIME2 (version 2020.06) and vegan, ggplot2, pheatmap, ggtree package in R (v3.6.0), which was carried out as described by Zhou et al. [25].

### 2.8. Statics Analysis

The statistical analyses were carried out using the software STATGRAPHICS Plus, version 4.0 (Copyright manugistics Inc., Rockville, MD, USA). Levels of significance (*p <* 0.05) of main treatments and their interactions were calculated by analysis of variance (ANOVA) after testing for normality and variance homogeneity.

## 3. Results and Discussion

The latest research in childhood neurodevelopmental disorders, including ASD, has transferred from genetics to environmental factors in the gut microbiome, which plays an important role [1,28,29]. Indeed, many studies have described the changes of intestinal microbiota of ASD children using non-culture-based methods [30,31,32]. This study investigated and compared the number, diversity, and community structure of gut bacteria from the fecal samples between healthy and ASD children using both the traditional plate counting method and 16S rRNA gene amplicon sequencing approaches. Furthermore, the effect of ZnONPs on gut bacteria were determined by a count of live cell number, living and dead bacterial cell staining and flow cytometry test, while the influence of ZnONPs in the composition of gut bacteria was determined by 16S rRNA gene sequence analysis of the obtained resistant strains in combination with 16S rRNA gene amplicon sequence analysis of fecal samples from healthy and ASD children.

### 3.1. Reduction of ASD and ZnONP in Cell Numbers by Plate Counting Method

**Figure 1:**
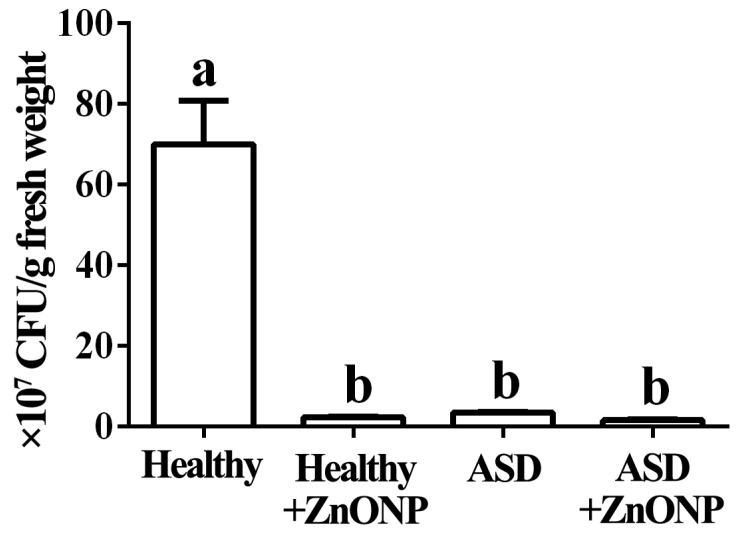
Cell numbers in the mixed fecal samples from the healthy or ASD children in the presence and absence of ZnONPs. Bacterial numbers were measured by the plate counting method. Vertical bars represent standard errors of the means (*n* = 6). Values in each column with different letters are significantly different (*p* ≤ 0.05).

The standard plate counting method results showed that the cell number of the live bacteria is 7.00 × 10^8^ CFU/g fresh feces in healthy children in the absence of ZnONP, while children with ASD caused a 94.96% reduction in the cell number of the live bacteria in fresh feces compared to the healthy children. Furthermore, there was a 96.64% reduction in the cell number of live bacteria in fresh feces from healthy children in the presence of ZnONP with a final density of 20 µg/mL, while there was a 97.64% reduction in the cell number of live bacteria in fresh feces from children with ASD compared to the healthy children in the presence of ZnONP with a final density of 20 µg/mL (Figure 1).

In agreement with the result of previous study [2], this study indicated that the numbers of the surviving gut bacteria in fresh feces from children with ASD were significantly less than that from healthy children. In addition, our result also found that the addition of ZnONPs resulted in a significant reduction in the cell number of gut live bacteria regardless of healthy or ASD children. Furthermore, no significant difference was observed in the cell number of gut live bacteria from ASD children between the presence and absence of ZnONPs, while no significant difference was found in the cell number of gut live bacteria between the healthy and ASD children in the presence of ZnONPs.

In agreement with the result of this study, Zhou et al. [25] found that the biosynthesized ZnONPs showed great antibacterial activity against gut live bacteria from healthy and ADHD children. However, compared to that of ADHD children, healthy children exhibited a more reduction in gut bacterial number. In contrast, ZnONPs in this study show similar inhibitory effect on the gut live bacteria from healthy and ASD children. To our knowledge, the result of this study firstly revealed that the ZnONPs had strong inhibitory effect on gut live bacteria from both healthy and ASD children by incubating the mixed fecal sample and then counting colony number on the nutrient agar medium.

### 3.2. Reduction of ASD and ZnONP in Bacterial Number by Living and Dead Cell Staining

The result of microscopic observation in this study showed that in the absence of ZnONP, there was a lot of live cells with membranes fluoresce bright green and dead cells with membranes fluoresce red-orange in mixed fresh feces of healthy children. Furthermore, in the presence of ZnONP at the final density of 20 µg/mL, a slight reduction was observed in the numbers of live gut bacteria, while no obvious change was observed in the numbers of dead gut bacteria in the mixed fresh feces of healthy children. This is a little different from the result of gut bacterial number using the plate counting method, which indicated that ZnONP had strong antibacterial activity against gut bacteria. This difference may be mainly due to the different methods used.

**Figure 2:**
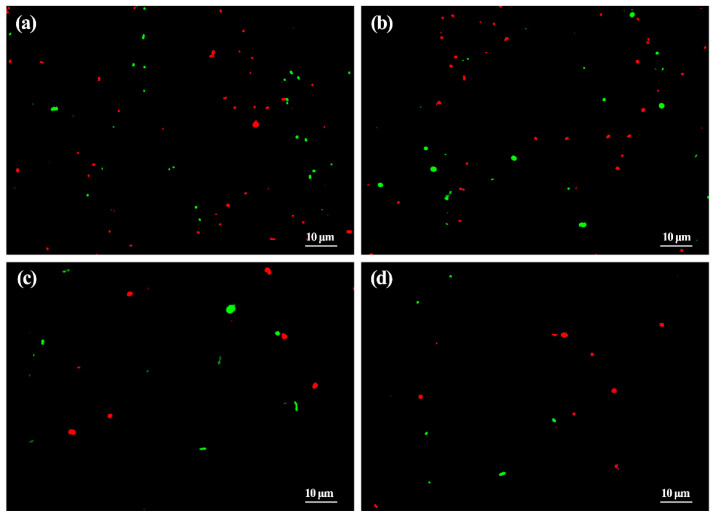
Antibacterial effect of ZnONP at the final density of 20 µg/mL on gut bacteria by living and dead cell staining: (**a**) healthy children; (**b**) healthy children + ZnONP; (**c**) ASD children; (**d**) ASD children + ZnONP.

This result of the live and dead bacterial cells staining also indicated that ASD children resulted in a significant (*p* < 0.05) reduction in the cell number of live and dead gut bacteria in fresh feces compared to the healthy children in both the presence and absence of ZnONP, which is consistent with the result of gut bacterial number based on the plate counting method. These results revealed that the reduction in live and dead bacterial cells by ASD children may be mainly due to the great inhibition in bacterial growth other than direct killing bacteria. Furthermore, the addition of ZnONP with a final density of 20 µg/mL caused a slight reduction in the cell number of live gut bacteria in the mixed fresh feces of ASD children compared to that in the absence of ZnONP (Figure 2). Although the live and dead bacterial cells staining reveals a general killing effect of gut bacteria, however, it is unclear about the killing effect of specific bacterial species.

### 3.3. Reduction of ASD and ZnONP in Bacterial Number by Flow Cytometry Test

The result of the flow cytometry tests indicated that there was a significant (*p* < 0.05) reduction in both cell number of gut bacteria and the live/dead ratio of gut bacteria in fresh feces of ASD children compared to that of the healthy children, indeed, 55.25% of total gut bacteria was alive and 44.75% of total gut bacteria was dead in healthy children, while 46.85% of total gut bacteria was alive and 53.15% of total gut bacteria was dead in ASD children in the absence of ZnONP. In contrast with the result of this study, Zhou et al. [25] found that in the absence of ZnONP, there was a similar live/dead ratio of gut bacteria in fresh feces between ADHD and healthy children, although the number of live bacteria in fresh feces from ADHD children was less than that of healthy children.

In agreement with the result of Zhou et al. [25], this result showed that ZnONP has a direct killing activity against gut bacteria. Indeed, the addition of ZnONP caused a significant (*p* < 0.05) increase in the dead percentage of gut bacteria regardless of heathy or ASD children. Indeed, there was a 38.67% and 61.33% of live and dead gut bacteria, respectively, in healthy children, while there was a 42.02% and 57.98% of live and dead gut bacteria, respectively, in ASD children in the presence of ZnONP. The addition of ZnONP resulted in a 37.05% increase in the ratio of dead to total gut bacteria from healthy children, which is similar to the result of Zhou et al. [25], who found that the addition of ZnONP caused a 37.53% increase in the ratio of dead to total gut bacteria in the mixed feces from healthy children.

**Figure 3:**
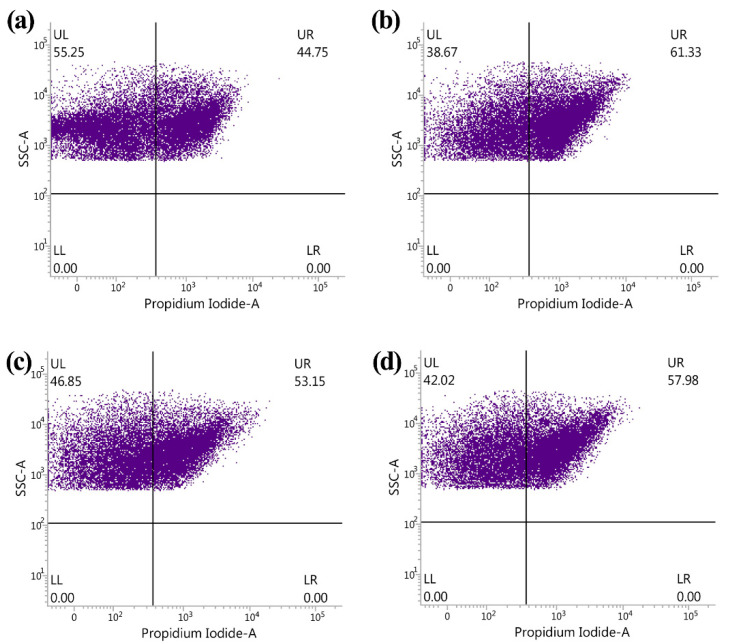
Antibacterial effect of ZnONP on gut bacteria from healthy and ASD children by flow cytometry observation: (**a**) healthy; (**b**) healthy + ZnONP; (**c**) ASD; (**d**) ASD + ZnONP.

However, the addition of ZnONP caused a 9.09% increase in the ratio of dead to total gut bacteria in the mixed feces from ASD children, which is obviously different from the result of Zhou et al. [25], who reported that the application of ZnONP resulted in a 39.15% increase in the ratio of dead to total gut bacteria in feces from ADHD children. This result showed that the gut bacteria in ASD children is generally more resistant to ZnONP than that of a healthy child. Furthermore, ASD children caused an 18.77% increase in the ratio of dead to total gut bacteria in the mixed feces compared to that from healthy children (Figure 3), while a greater reduction in gut bacterial number was observed in both the plate counting method and live-dead staining. This conflict result may be mainly due to the different method used. In addition, it is more convenient for us to examine bacterial viability using the method of living and dead cell staining than that of the plate counting method. However, the former required completely removing the impurities from fecal samples.

### 3.4. Isolation and Identification of Gut Bacteria with ZnONPs Resistantance

The main bacterial species resistant to ZnONPs were collected by inoculating the fecal samples supplement with ZnONP at the final density of 20 µg/mL on the NA medium and then picking up the main representative isolates that were selected by the shape, color and edge of bacterial colony. Furthermore, the representative bacteria from the fecal sample of healthy children were usually clustered into three different groups, while the representative bacteria from the fecal sample of the ASD children were clustered into one same group based on the analysis of 16S rDNA gene sequence. Indeed, the four representative strains 6-1, 6-2, 6-3 and 6-4 from healthy children were identified as *Bacillus anthracis*, *Escherichia coli*, *Bacillus cereus* and *Escherichia coli*, respectively, with 97.86%, 99.86%, 99.03% and 99.65% sequence similarity of 16S rRNA gene, while the three representative strains 8-1, 8-2 and 8-3 from ASD children were identified as *Bacillus cereus*, with 99.58%, 99.72% and 99.72%, respectively, sequence similarity of 16S rRNA gene (Table 1).

Interestingly, we also noted that a high ratio of ZnONPs-resistant gut bacteria was Gram positive. In agreement with the result of this study, previous studies have shown that nanoparticles exhibit a strong antibacterial effect against plant, animal and human pathogenic bacteria in particular those are Gram negative [33,34]. Gram-positive bacteria have thick peptidoglycan layers, which was able to protect themselves against antimicrobial agents. In contrast, the negative charge of the lipopolysaccharide layer in Gram-negative bacteria facilitates the adsorption of positively charged nanoparticles [33,34]. Some other researchers have also confirmed that wall thickness plays an important role in bacterial resistance to nanoparticles [35,36].

The present study revealed that the application of ZnONPs was able to cause the reconstruction of gut bacterial community structure. Indeed, there was a great difference between healthy and ASD children in the main gut bacterial structure that resistance to ZnONPs. Indeed, there was a greater diversity for ZnONPs-resistant gut bacteria in fecal samples from healthy children compared that from the ASD children. This difference may be mainly due to the change of ASD children in bacterial community structure compared to healthy children. Similarly, Zhou et al. [25] reported the change of ZnONPs in gut bacterial community structure in ADHD children.

In order to give a comprehensive assay in community structure of gut bacteria from healthy and ASD children, some previous studies have also investigated the general gut microbiota population in ASD children using the non-culture method [37,38,39]. However, the main bacterial community varied in different studies, revealing the complexity of gut bacteria in healthy and ASD children. As we know, the non-culture method exhibits the disadvantage of being unable to clear whether the gut bacteria is alive or dead, although it has the advantage of quicker results. Interestingly, using the culture method, this study provides an alternate to investigate the gut microbiota by focusing on the live bacteria in fecal samples from heathy and ASD children. In particular, this study successfully obtained some gut bacteria that showed great resistance to ZnONPs. However, isolation of gut bacteria in this study was carried out on nutrient agar medium, which may cause a loss of some bacteria that have specific growth requirements.

### 3.5. Effect of ZnONPs on Community Diversity of Gut Bacteria

**Figure 4:**
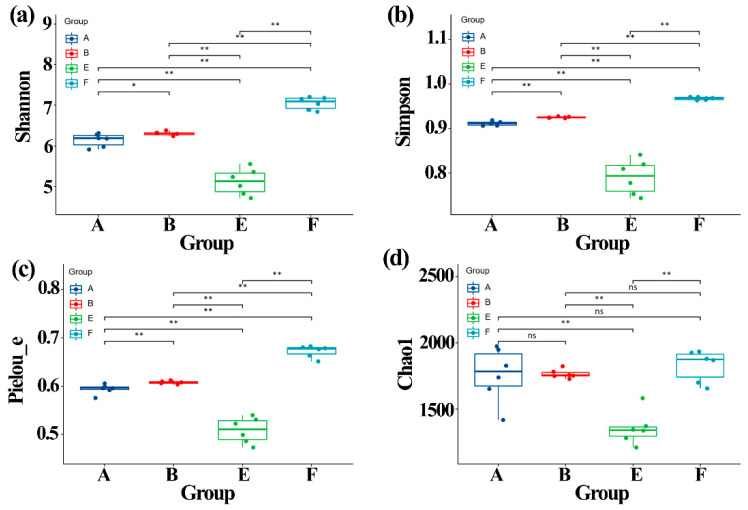
Effect of ZnONP on the alpha diversity of gut bacteria. (**a**) Shannon index; (**b**) Simpson index; (**c**) Pielou_e index; (**d**) Chao1. A: healthy children; B: healthy children + ZnONP; E: ASD children; F: ASD children + ZnONP. *: 0.05; **: 0.01; ns: not significance.

The alpha diversity of gut bacteria was determined in this study based on 16S rRNA amplicon sequence analysis, which was performed by calculating and comparing the Chao1, Shannon, Simpson and Pielou_e indexes after normalizing each data to the same read number (37,350 sequences, the smallest sequence number). As shown in Figure 4, the four different indexes of community diversity was significantly (*p* < 0.01) lower in gut bacteria from ASD children than that from healthy children. However, in contrast with the result of this study, Zhou et al. [25] reported that the three different indexes of the community diversity was significantly (*p* < 0.01) higher in gut bacteria from ADHD children than that from healthy children.

Interestingly, the results of this study also revealed that the biosynthesized ZnONPs had an influence in the community diversity index of gut bacteria from both healthy and ASD children. Indeed, Chao1 index was unaffected by the addition of ZnONP, which caused a significant increase in Shannon (*p* < 0.05), Simpson (*p* < 0.01) and Pielou_e (*p* < 0.01) indexes of gut bacteria from healthy children. In addition, the addition of ZnONP resulted in a significant (*p* < 0.01) increase in the four different indexes of community diversity. Furthermore, the increase of ZnONP in the four different community diversity indexes in ASD children is greater than that in healthy children (Figure 4).

Generally, this study showed that the community diversity of gut bacteria was reduced by ASD, which is consistent with the reduction of ASD in cell numbers of gut bacteria compared to healthy children. In contrast, the ZnONPs significantly increased the gut bacterial community diversity. Furthermore, in agreement with the result of Zhou et al. [25], the community diversity of gut bacteria in healthy children was increased by the ZnONPs, which is different with the change in cell numbers of gut bacteria. However, the increase of the ZnONPs in gut bacterial community diversity in ASD children is different from the result of Zhou et al. [25], which reported that ZnONPs caused a significant reduction in gut bacterial community diversity in ADHD children.

**Figure 5:**
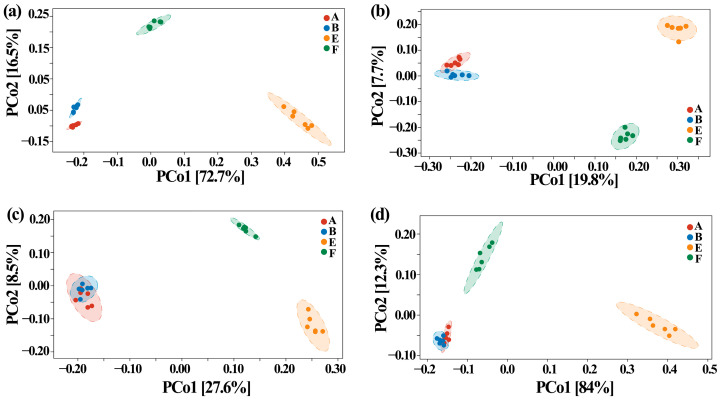
Effect of ZnONP on beta diversity of gut bacteria by PCoA of (**a**) Bray–Curtis, (**b**) Jaccard, (**c**) unweighted UniFrac and (**d**) weighted UniFrac. A: healthy children; B: healthy children + ZnONP; E: ASD children; F: ASD children + ZnONP.

Furthermore, the influence of ZnONP on the beta diversity of gut bacteria in fecal samples from healthy and ASD children was assayed by using principal coordinates analysis (PCoA) at ASVS level with Bray–Curtis, Jaccard, unweighted UniFrac and weighted UniFrac. In general, the PCoA of the four indicators indicated that there was a 7.7–84.0% variation of bacterial community composition among the tested fecal samples. In detail, there was a 72.7%, 19.8%, 27.6% and 84.0% variation between healthy and ASD children based on PCo1 of Bray–Curtis, Jaccard, unweighted UniFrac and weighted UniFrac parameters, respectively, while there was a 16.5%, 7.7%, 8.5% and 12.3% variation between with and without ZnONPs based on PCo2 of Bray–Curtis, Jaccard, unweighted UniFrac and weighted UniFrac parameters, respectively (Figure 5).

**Figure 6:**
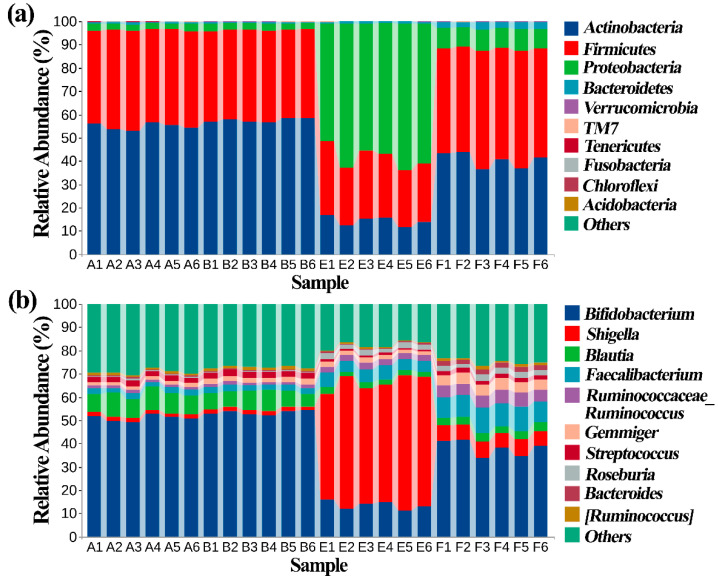
Assay of hierarchical clustering constructed at the levels of (**a**) phylum and (**b**) genus according to Bray–Curtis dissimilarity (*n* = 6). A: healthy children; B: healthy children + ZnONP; E: ASD children; F: ASD children + ZnONP.

Similarly, Zhou et al. [25] revealed that the gut bacterial community structure varied between the healthy and ADHD children based on the above-mentioned four different indicators. Therefore, it can be inferred that the above-mentioned four indicators were able to be used to differentiate the difference in gut bacterial community structure among the samples tested in this study (Figure 5). Furthermore, this result also revealed that compared to the nanoparticles, ASD exhibited a greater influence in gut bacterial community structure in term of the analysis of hierarchical clustering, which was constructed at the levels of phylum and genus according to the Bray–Curtis dissimilarity (Figure 6). This data is consistent with Zhou et al. [25], who found that ADHD had a greater differential effect than that of nanoparticles. However, further studies should be carried out to include more fecal samples from ASD children.

### 3.6. Effect of ZnONPs on Species Composition of Gut Bacteira

**Figure 7:**
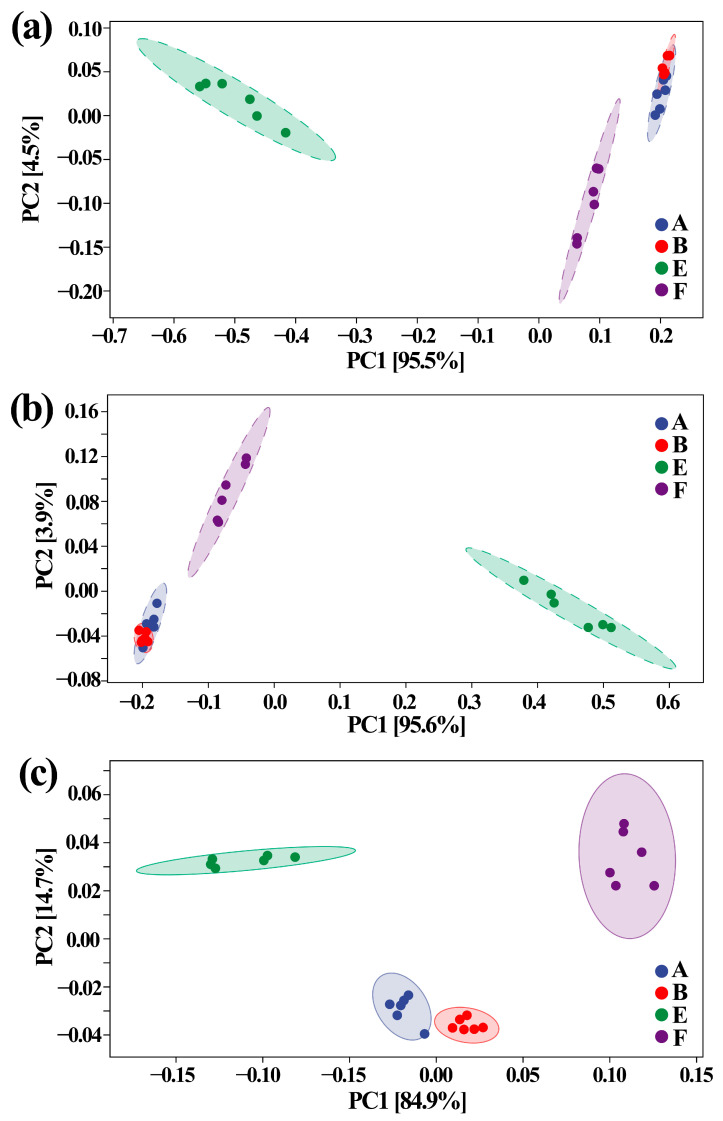
PCA of gut bacteria at levels of (**a**) phylum, (**b**) genus and (**c**) species. A: healthy children; B: healthy children + ZnONP; E: ASD children; F: ASD children + ZnONP.

Effect of ZnONPs on species composition of gut bacteria was determined by 16S rRNA amplicon sequencing analysis, which was carried out in six replicates of each sample according to PCA at the levels of phylum, genus and species. The result of this study revealed that there was an overall similarity in gut bacterial community structure within each sample. However, different samples differed in the gut bacterial community structure. Furthermore, in agreement with the diversity analysis result, ASD caused a greater change of gut bacterial community structure compared to the nanoparticles. Indeed, there was a 95.5%, 95.6% and 84.9% total variation between healthy and ASD children in PC1 at levels of phylum, genus, and species, respectively, while there was a 4.5%, 3.9% and 14.7% total variation between in presence and absence of nanoparticles in PC2 at levels of phylum, genus, and species, respectively, based on the result of PCA (Figure 7).

The comparison of this data with the greengene database indicated that the top 10 phylum of gut bacteria among the tested samples was *Actinobacteria*, *Firmicutes*, *Proteobacteria*, *Bacteroide*, *Verrucomicrobia*, TM7, *Tenericutes, Fusobacteria*, *Chloroflexi*, and *Acidobacteria*. There was no difference in healthy children between with and without nanoparticles in the taxonomic composition of gut bacteria, of which *Actinobacteria* (56.2%) and *Firmicutes* (40.1%) were the main groups. However, there was a great difference in ASD children between with and without nanoparticles in the taxonomic composition of gut bacteria, indeed, *Actinobacteria* (14.26%), *Firmicutes* (27.9%) and *Proteobacteria* (57.6%) were the main groups in the former, while *Actinobacteria* (40.4%), *Firmicutes* (47.7%), *Proteobacteria* (8.8%) were the main groups in the latter.

**Figure 8:**
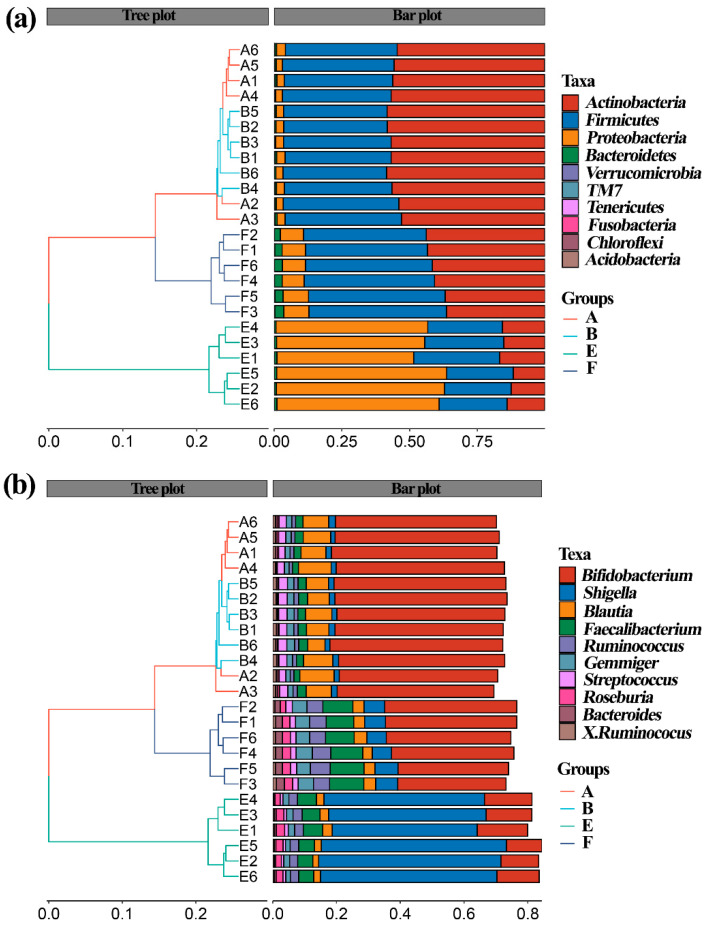
Effect of ZnONP on classification and composition diagram of gut bacteria at levels of (**a**) phylum and (**b**) genus. A: healthy children; B: healthy children + ZnONP; E: ASD children; F: ASD children + ZnONP.

Similarly, no obvious difference was observed in the taxonomic composition of gut bacteria in healthy children in between presence and absence of nanoparticles at the genus level, while there was a great difference in the taxonomic composition in ASD children between with and without nanoparticles at the genus level. For example, there were 52.6% and 13.6% relative abundances of *Shigella* and *Bifidobacterium*, respectively, in ASD children in the absence of nanoparticles. In contrast, in the presence of nanoparticles, the two genera in ASD children were 6.5% and 38.1%, respectively (Figure 8). In addition, the taxonomic composition in healthy children differed with that of ASD children regardless of the presence or the absence of nanoparticles.

**Figure 9:**
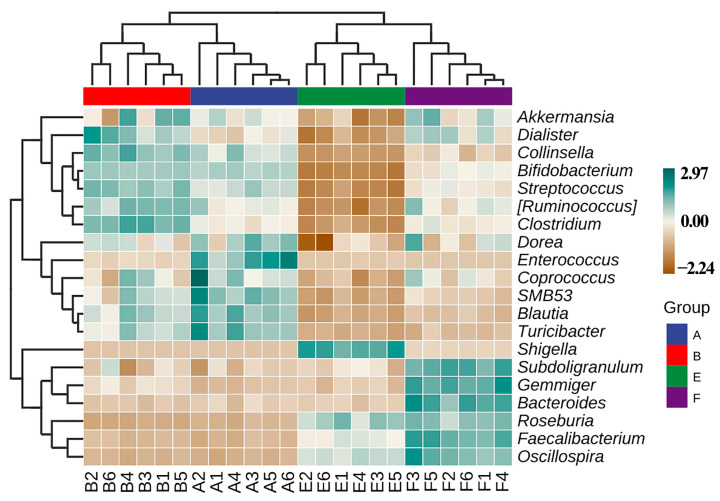
Effect of ZnONP on composition and distribution of the main 20 genus among samples. A: healthy children; B: healthy children + ZnONP; E: ASD children; F: ASD children + ZnONP.

Effect of ZnONP on species composition of gut bacteria from ASD and healthy children in the presence and absence of ZnONP based on the heat map, which was drawn based on the average abundance data of the top 20 genera. This study found that there was a significant change in the gut microbial community structure between healthy and ASD children (Figure 9), which is consistent with the data of previous studies [40,41,42]. Indeed, *Enterococcus, SMB53, Blautia* and *Turicibacter* are enriched in healthy children in absence of nanoparticles, while *Collinsella,Ruminococcus* and *Clostridium* are enriched in healthy children in presence of nanoparticles. Furthermore, *Shigella* was enriched in ASD children in the absence of nanoparticles, while *Subdoligranulum, Gemmiger, Bacteroides,Roseburia,Faecalibacterium, Oscillospira* were enriched in ASD children in the presence of nanoparticles.

Obviously, the result of this study revealed the differences in the trend of gut bacterial species abundance distribution, which was affected by both the ASD and the nanoparticles. However, the conflict results were reported in various studies, for example, Ding et al. [31] found that there was a significant increase in the relative abundance of unidentified *Lachnospiraceae*, *Clostridiales*, *Erysipelotrichaceae*, *Dorea*, *Collinsella* and *Lachnoclostridium*, while *Bacteroides*, *Faecalibacterium*, *Parasutterella* and *Paraprevotella* were significantly lower in the ASD group than in the control group. Bezawada et al. [8] revealed that species reported to be significantly higher in abundance in autistic children included *Clostridium*, *Sutterella*, *Desulfovibrio* and *Lactobacillus*. Iglesias-Vázquez et al. [37] reported that ASD children exhibited a significantly higher abundance of the genera *Clostridium*, *Parabacteroides*, *Phascolarctobacterium*, *Faecalibacterium*, *Bacteroides* and a lower percentage of *Bifidobacterium* and *Coprococcus*. In contrast, Ahmed et al. [43] reported that the gut microbiome of ASD children and their siblings contained a higher relative abundance of *Bacteroides* than controls. Interesting, only one genus *Shigella* was enriched in ASD children, thus, it can be inferred that this genus may be highly associated with the development of ASD. Fortunately, the addition of nanoparticles resulted in the disappearance of *Shigella* from the enriched genus of gut bacteria from ASD children, suggesting that nanoparticles may have a great potential in the prevention of ASD.

In order to open the possibility for new potential strategies in this disease, it is very necessary to identify ASD-associated gut bacteria, however, very limited scientific investigation into the potential biological connection between the ASD and gut bacteria. In particular, different studies often showed the conflicted results in this field, for example, Ding et al. [31] revealed that the presence of unidentified *Faecalibacterium*, *Erysipelotrichaceae*, and *Lachnospiraceae* was correlated with the severity of ASD. Ahmed et al. [43] found that there was no correlation between the severity of autism symptoms and the altered gut microbiome, although evidence of changes was revealed in the gut microbiome of autistic children compared to the unrelated control. However, Andreo-Martínez et al. [32] recently found that it did not show evidence of a relevant gut microbiome-ASD association for *Proteobacteria*, *Actinobacteria Firmicutes* and *Bacteroidetes* phyla based on the meta-analysis integrated effect sizes of 18 previous studies to assess the potential association between gut microbiota and ASD.

In general, the result of this study indicated that ASD was able to result in the reduction in both gut bacterial number and bacterial community diversity. Interestingly, previous studies have reported that there was a high comorbidity between ASD and ADHD individuals [43]. However, the change of ASD children in bacterial community diversity is different from that of ADHD children [25]. This can be justified by the result of different social behavior between ASD and ADHD. On the other hand, the inconsistent findings in the association between gut microbiota and ASD can be explained partially by heterogeneous populations and methods. Furthermore, it has been well known that the gut microbiota was affected by various factors, such as diet and antibiotic treatment. Therefore, in order to provide more evidences to clarify the role of gut bacteria in the development of ASD, details regarding antibiotic use, dietary habits, living conditions, host genetic factors and clinical assessment are important to revealing the link between autism and an altered gut microbiota.

## 4. Conclusions

In the present study, we successfully isolated, identified and characterized the gut bacteria partially nanoparticle-resistant bacteria from the mixed fecal samples of healthy and ASD children in China by the combination of the traditional plate method with amplicon sequence analysis. The result not only revealed significant changes in the cell number, diversity and community composition of gut bacteria from the mixed fecal samples of ASD children compared to the healthy children, but also found that ZnONPs have a differential effect in number, diversity and community composition of gut bacteria from healthy and ASD children. In particular, only one genus *Shigella* was found to be enriched in the ASD children, but this genus disappeared from the enriched genus in the presence of ZnONPs. In conclusion, this study demonstrated that ZnONPs changed the number, diversity, and species composition of gut bacteria, but differed in healthy and ASD children.

## Figures and Tables

**Table 1:** Similarity analysis of 16S rDNA sequence in the representative bacterial strains from the mixed fecal samples of the ASD and healthy children.

Bacterial Strains	Sources	Bacterial Identity (Sequence Similarity of 16S rDNA)	Accession No.
Strain 6-1	Healthy + ZnONP	*Bacillus anthracis* (97.86%)	MW664950
Strain 6-2	Healthy + ZnONP	*Escherichia coli* (99.86%)	MW664951
Strain 6-3	Healthy + ZnONP	*Bacillus cereus* (99.03%)	MW664952
Strain 6-4	Healthy + ZnONP	*Escherichia coli* (99.65%)	MW664953
Strain 8-1	ASD + ZnONP	*Bacillus cereus* (99.58%)	MW664954
Strain 8-2	ASD + ZnONP	*Bacillus cereus* (99.72%)	MW664955
Strain 8-3	ASD + ZnONP	*Bacillus cereus* (99.72%)	MW664956

## Data Availability

All data supporting the conclusions of this article are included in this article. The 16S rDNA gene sequences of the representative strains 6-1, 6-2, 6-3, 6-4, 8-1, 8-2 and 8-3 have been deposited at GenBank database with Accession No. MW664950- MW664956.
